# Bilateral Trochleoplasty Procedures Following Bilateral Femoral Rotational Osteotomies for Bilateral Dejour Type D Trochlear Dysplasia

**DOI:** 10.31486/toj.25.0066

**Published:** 2026

**Authors:** Laura Linscheid, Jonathan Willard, Eden Schoofs, W. Evans Few, Deryk Jones

**Affiliations:** ^1^ Ochsner Andrews Sports Medicine Institute, Ochsner Clinic Foundation, New Orleans, LA; ^2^ Department of Emergency Medicine, Ochsner Clinic Foundation, New Orleans, LA; ^3^ The University of Queensland Medical School, Ochsner Clinical School, New Orleans, LA

**Keywords:** *Joint instability*, *knee joint*, *patella*, *patellar dislocation*, *trochlear dysplasia*, *trochleoplasty*

## Abstract

**Background:**

Trochlear dysplasia is a considerable risk factor for patellar instability. Surgical treatment options include medial patellofemoral ligament reconstruction, medial quadriceps tendon–femoral ligament reconstruction, femoral rotational osteotomy, tibial tubercle osteotomy, and trochleoplasty.

**Case Report:**

A 26-year-old female presented with bilateral knee pain and recurrent patellar dislocations since childhood that were unresponsive to physical therapy and bracing. Initial evaluation by an orthopedic trauma service revealed excessive femoral anteversion and Dejour type D trochlear dysplasia bilaterally. A right femoral rotational osteotomy with intramedullary nail fixation improved right-sided patellar stability. Eighteen months after the first surgery, the patient underwent a planned left femur rotational osteotomy. The patient continued to experience recurrent bilateral patellar subluxation and was referred for definitive treatment. Examination demonstrated bilateral patellar subluxation at rest and positive J-sign on flexion, with the left knee more symptomatic than the right. Imaging revealed healed osteotomies, persistent Dejour type D trochlear dysplasia, and elevated tibial tubercle–trochlear groove distances bilaterally. Merchant view radiographs and magnetic resonance imaging showed substantial lateral patellar subluxation on the left with intact patellofemoral cartilage. Computed tomography confirmed bilateral trochlear dysplasia. The patient underwent left knee recession wedge trochleoplasty, tibial tubercle osteotomy, and medial quadriceps tendon–femoral ligament reconstruction. One year later, the same procedures were performed on the right knee with good results.

**Conclusion:**

Trochleoplasty, tibial tubercle osteotomy, and medial quadriceps tendon–femoral ligament reconstruction are effective when performed concomitantly for recalcitrant patellar instability associated with trochlear dysplasia and failed prior surgical intervention.

## INTRODUCTION

Patellar instability is a common orthopedic condition with symptoms that range from subluxation to complete dislocation.^1-3^ Patellar dislocation has a prevalence of approximately 5.8 per 100,000 patients.^4^ Multiple anatomic factors predispose patients to patellar instability, such as ligamentous laxity, excessive femoral anteversion, patella alta, and trochlear dysplasia.^5^

In a 1994 radiographic study of 143 knees, trochlear dysplasia was identified as a relevant factor in patellar instability, with trochlear dysplasia present in approximately 85% of patients with symptomatic patellar instability.^6^ Trochlear dysplasia refers to abnormal bony morphology of the distal femur affecting the trochlear groove, most commonly characterized by a shallow and flattened articulation. On axial imaging, the groove is often shallow yet concave, with associated lateral facet convexity and medial facet hypoplasia; in more severe cases, the trochlea may appear dome-shaped or effectively convex.^7^ This lack of stability typically creates lateral subluxation or dislocation of the patella, leading to tearing and insufficiency of the medial patellofemoral ligament and medial quadriceps tendon–femoral ligament structures.^8,9^ The Dejour classification, which categorizes trochlear dysplasia into 4 types (A to D) based on the presence of the crossing sign, supratrochlear spurs, and a double contour sign on radiographic imaging, is a widely accepted method for characterizing trochlear dysplasia.^10,11^ Fucentese et al classified trochlear dysplasia types as moderate (DeJour types A and C) or severe (DeJour types B and D) and found that trochleoplasty was a useful and reliable technique for improving patellofemoral instability.^12^ The Fucentese et al study showed that patients with DeJour types B and D trochlear dysplasia had significantly better clinical outcomes after trochleoplasty compared to patients with DeJour types A and C.^12^

Primary patellar dislocation is a common orthopedic condition, accounting for 3% of all knee injuries.^2^ Up to 40% of patients with primary patellar dislocation may have recurrent dislocations with a corresponding decrease in function and quality of life.^13^ Mitchell et al described young, active females to be most at risk for patellofemoral instability injuries.^14^ Anatomic abnormalities to be considered include trochlear dysplasia; patella alta; a high tibial tubercle–trochlear groove distance; weakness of the vastus medialis obliquus; and tearing of the medial patellofemoral ligament, medial quadriceps tendon–femoral ligament, and medial patellar retinaculum.^5,15^ Summarizing the findings from the literature, Pascual-Leone et al highlighted anatomy, demographic factors, and medical history as predictive for recurrent injury after primary dislocation.^16^ Depending on anatomic abnormalities and medical history, surgical management of patellar instability in the setting of trochlear dysplasia may include trochleoplasty, rotational osteotomies, tibial tubercle osteotomy, and ligament reconstruction.

We present the case of a 26-year-old female treated with bilateral trochleoplasty, tibial tubercle osteotomy, and medial quadriceps tendon–femoral ligament reconstructions for recurrent patellar instability in the setting of bilateral Dejour type D trochlear dysplasia after bilateral femoral rotational osteotomies.

## CASE REPORT

**Figure 1.**
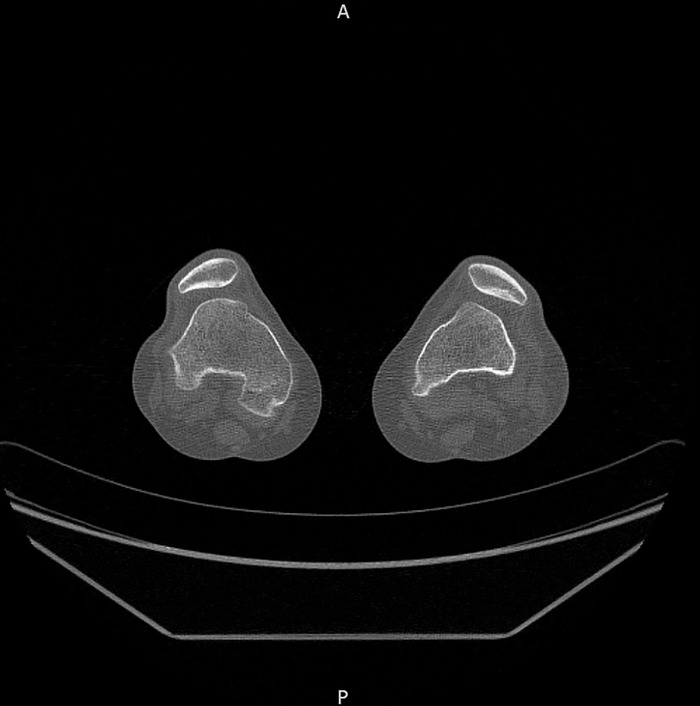
Preoperative axial computed tomography scan of the knees shows bilateral excessive femoral anteversion measuring 25° on the right and 24° on the left. The tibial tubercle–trochlear groove distances are 21 mm on the right and 25 mm on the left.

**Figure 2.**
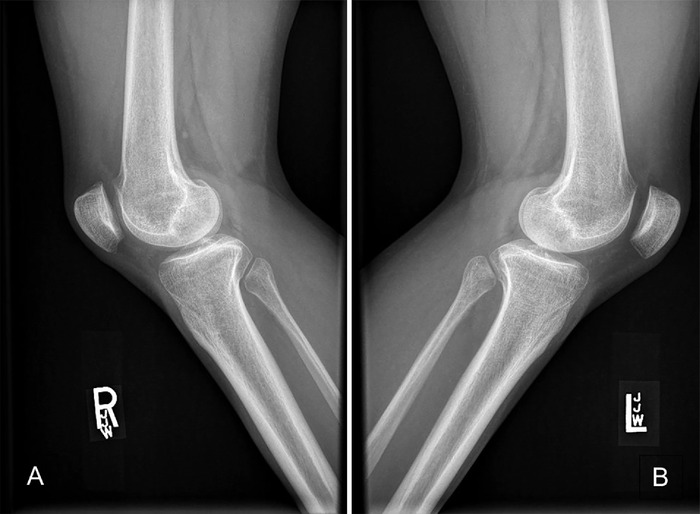
Preoperative lateral radiographic images show (A) right knee with a Caton-Deschamps index of 1.27 and (B) left knee with a Caton-Deschamps index of 1.20.

**Figure 3.**
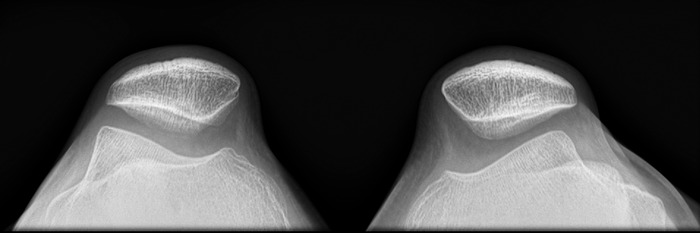
Merchant view radiograph shows left greater than right lateral patellar subluxation at 45° flexion.

**Figure 4.**
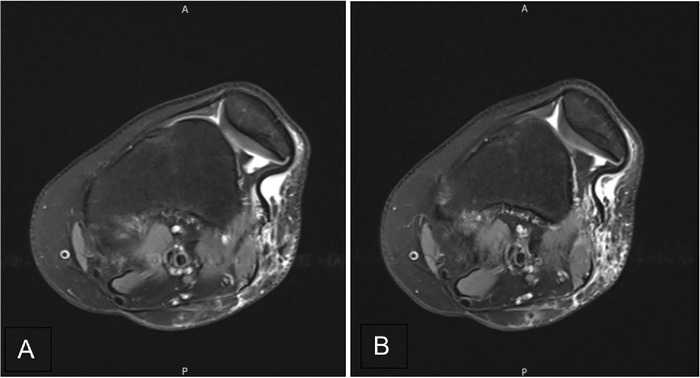
(A, B) Preoperative magnetic resonance imaging of the left knee with cartilage-specific sequences shows severe lateral patellar subluxation but minimal articular cartilage damage.

A 26-year-old female with a medical history of hyperthyroidism initially presented with bilateral knee pain and recurrent patella dislocations since childhood. The patient had tried physical therapy and patellar stabilizing braces without relief. Imaging showed excessive femoral anteversion measuring 25° on the right and 24° on the left. The tibial tubercle–trochlear groove distances were 21 mm on the right and 25 mm on the left (Figure 1). Caton-Deschamps indices were 1.27 on the right and 1.20 on the left (Figure 2). Merchant view radiograph showed left greater than right lateral patellar subluxation at 45° flexion (Figure 3). Magnetic resonance imaging (MRI) of the left knee with cartilage-specific sequences showed severe lateral patellar subluxation but minimal articular cartilage damage (Figure 4).

At a separate hospital within the same integrated health system, the initial treating surgeon performed a right femur rotational osteotomy with intermedullary nail fixation. This procedure stabilized the right patellofemoral joint relative to the left knee, but the patient continued to experience bilateral patellar instability. Eighteen months after the first surgery, the patient underwent a planned left femur rotational osteotomy performed by the initial surgeon at the same hospital.

**Figure 5.**
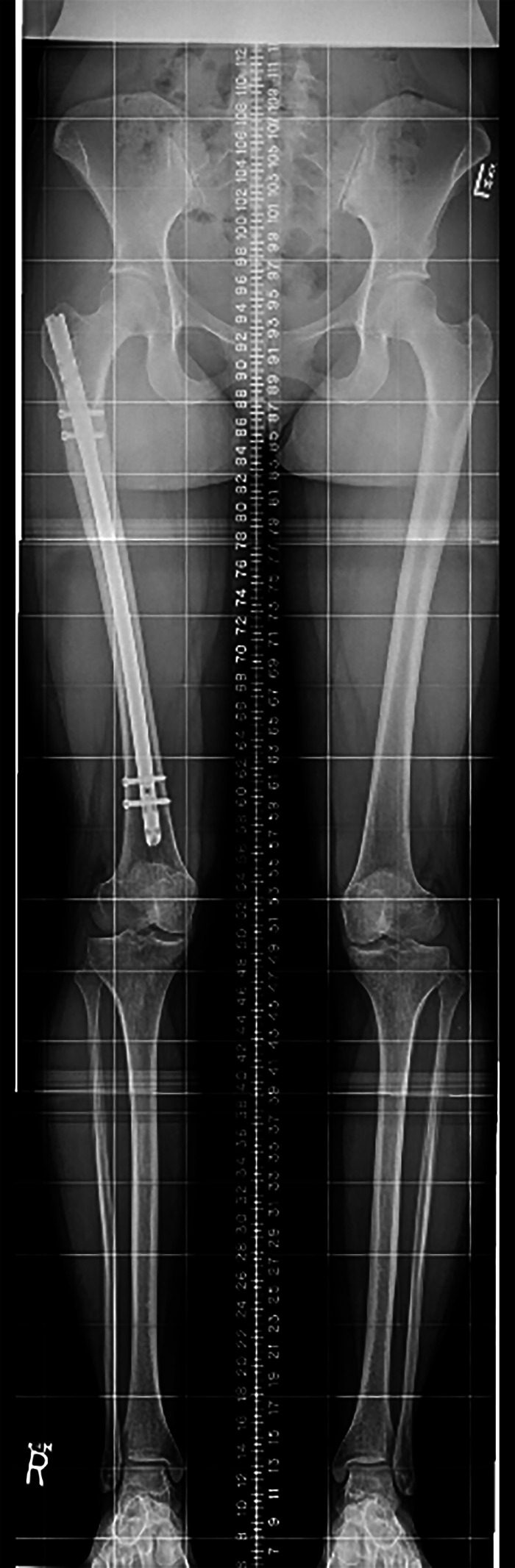
Hip to ankle long leg standing radiograph shows healed right femoral osteotomy with 2° valgus and left knee neutral alignment.

Despite the bilateral femoral rotational osteotomies, the patient continued to experience recurrent bilateral patellar subluxation and was referred to the senior author for definitive treatment. Approximately 7 months following the left femur rotational osteotomy, the patient was seen in clinic by the senior author and reported continued pain and patellar subluxations on the left greater than the right. Patient-reported outcome measures (PROMs) were recorded at this initial clinic visit (Table 1). Physical examination at this time demonstrated bilateral patellar subluxations, J-signs, and painful effusions. The full workup at this time revealed a healed femoral osteotomy on the right knee with 2° valgus and left knee neutral alignment on hip to ankle long leg standing radiographs (Figure 5). The bilateral femoral osteotomies were allowed to heal but because of continued bilateral lateral patellar subluxations, further attention was placed on the bilateral trochlear dysplasia. Because the patient reported predominant left knee symptomatology and left worse than right PROMs, the decision was made to proceed with left knee diagnostic arthroscopy, recession wedge trochleoplasty, tibial tubercle osteotomy, and medial quadriceps tendon–femoral ligament reconstruction. The senior author performed the surgery 11 months after the patient's referral visit. PROMs were recorded at the patient's preoperative visit 2 weeks prior to the procedure (Table 1).

**Table 1.** Patient-Reported Outcome Measures for the Left Knee

					Knee injury and Osteoarthritis Outcome Score (KOOS)					Short Form Health Survey	
Time Point	Pain Frequency^a^	Pain Severity^b^	IKDC Function^c^	Lysholm^d^	Pain^e^	Symptom^f^	ADL^g^	Sport^h^	QoL^i^	PSF-12^j^	MSF-12^k^
Initial visit	2	5	62.06	60	80.56	54.29	94.12	75	25	41.7	59.05
2 weeks preoperatively	4	4	59.77	52	66.67	60.71	91.18	50	12.5	39.86	61.94
6 weeks postoperatively	8	6	29.88	51	63.89	57.14	39.71	10	12.5	23.42	57.02
3 months postoperatively	7	2	45.97	63	63.89	57.14	77.94	50	56.25	34.64	54.92
6 months postoperatively	5	6	51.72	76	66.67	57.14	92.65	65	50	31.49	60.73
14 months postoperatively	0	0	100	100	100	100	100	100	100	34.97	60.52
23 months postoperatively	1	1	95.4	94	94.44	82.14	100	100	87.5	53.8	57.92

^a^Pain frequency was subjectively scaled from 0 to 10, with 0 being never and 10 being constant.

^b^Pain severity was subjectively scaled from 0 to 10, with 0 being no pain and 10 being the most severe pain.

^c^The International Knee Documentation Committee (IKDC) form is scored from 0 to 100, with higher scores indicating better knee function and lower symptoms. A score of 100 indicates no limitations.

^d^The Lysholm Knee Scoring Scale is scored from 0 to 100, with higher scores indicating better knee function and fewer symptoms. It is an 8-item, patient-reported outcome measure assessing limp, support, locking, instability, pain, swelling, stair climbing, and squatting, with scores typically categorized as poor (<65), fair (65-83), good (84-94), and excellent (95-100).

^e^The KOOS pain outcome is scored from 0 to 100, with higher scores indicating better function and less knee-related pain.

^f^The KOOS symptom outcome is scored from 0 to 100, with higher scores indicating better function and fewer knee-related symptoms.

^g^The KOOS activities of daily living (ADL) outcome is scored from 0 to 100, with higher scores indicating better function and fewer knee-related problems with activities of daily living.

^h^The KOOS sport outcome is scored from 0 to 100, with higher scores indicating better function and fewer knee-related problems with sporting activities.

^i^The KOOS quality of life (QoL) outcome is scored from 0 to 100, with higher scores indicating better function and fewer knee-related problems impacting quality of life.

^j^The Short Form Health Survey physical component score (PSF-12) is scored from 0 to 100, with higher scores indicating better physical health status. Scores are norm-based (mean of 50, standard deviation of 10 in the general population), with values above or below 50 representing better or worse health relative to population norms.

^k^The Short Form Health Survey mental component score (MSF-12) is scored from 0 to 100, with higher scores indicating better mental health status. Scores are norm-based (mean of 50, standard deviation of 10 in the general population), with values above or below 50 representing better or worse health relative to population norms.

### Operative Technique

**Figure 6.**
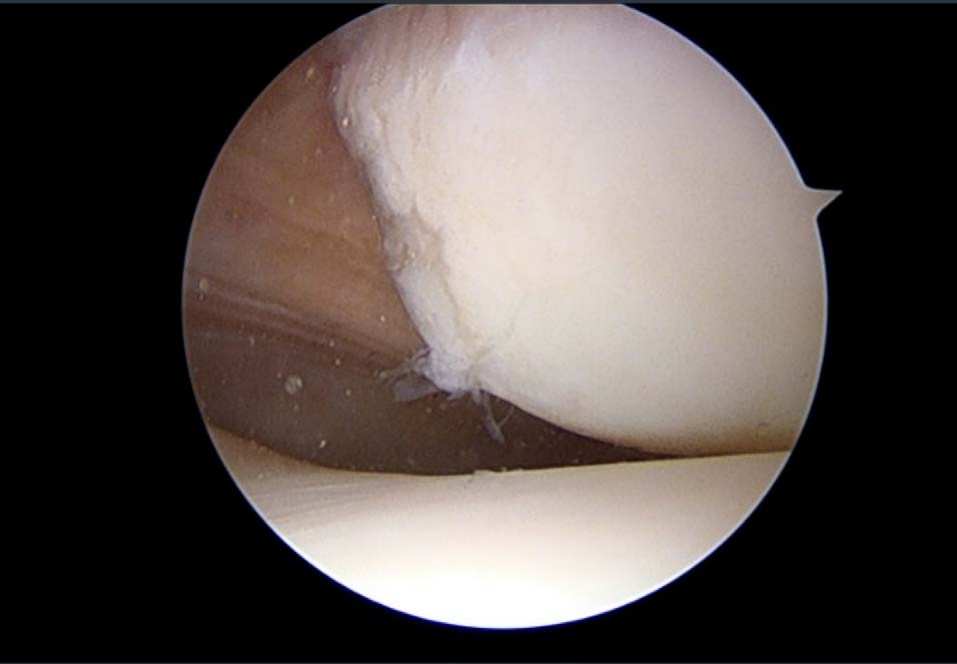
Arthroscopic image shows lateral patellar subluxation, flattened trochlear groove, and retained articular cartilage with minimal softening noted on probe analysis.

**Figure 7.**
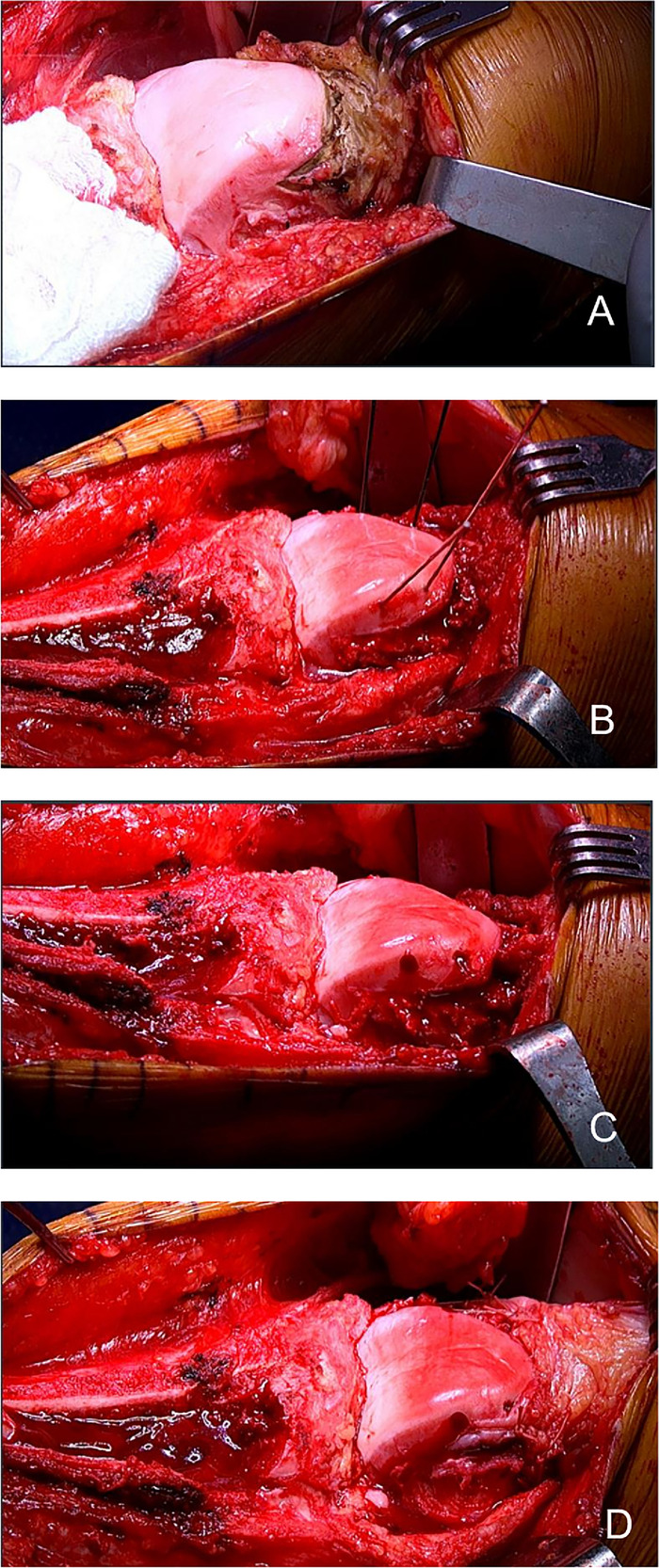
Intraoperative photographs show (A) the suprapatellar spur and retracted synovium, (B) pin placement, (C) screw placement, and (D) synovium allowed to retract distally and sewed with #1 Vicryl sutures (Johnson & Johnson MedTech).

Diagnostic arthroscopy showed severe lateral patellar tilt and subluxation, trochlear dysplasia, and retained patellofemoral articular cartilage (Figure 6). The rest of the knee evaluation was normal. Tibial tubercle osteotomy was performed with a Hall MicroFree sagittal cordless saw (CONMED Corporation) utilizing a MD3T guide system (Kinamed, Inc). This system was used to create a primary wedge and a 10-mm secondary wedge. Lateral retinacular lengthening was performed, leaving the entire vastus medialis obliquus attached to the patella. Lengthening of the lateral patellar retinaculum allowed full exposure of the trochlear region. The supratrochlear spur was visualized by carefully elevating a flap of synovial tissue from the anterior distal femoral region proximal to the trochlea; the area of wedge recession was demarcated based on anatomic assessment of the bony anatomy (Figure 7A). The same saw blade was used to create a wedge-shaped resection from the proximal trochlear base with a thickness of 6 mm proximally and tapering to 0 mm distally. Plastic deformation was created at the distal wedge resected region by applying a posterior directed force; the periphery of the trochlea was pinned with 4 guidewires (Figure 7B). Sequential placement of 4 Acutrak 2.5-mm screws (Acumed LLC) with the countersink technique was performed with lengths 30 mm proximally and 28 mm distally. Screws were placed peripherally, avoiding extension into the central articular cartilaginous region (Figure 7C). The elevated synovial flap was then allowed to retract back into the original position distally and was sewn with a series of figure-of-8 #1 Vicryl sutures (Johnson & Johnson MedTech) (Figure 7D).

**Figure 8.**
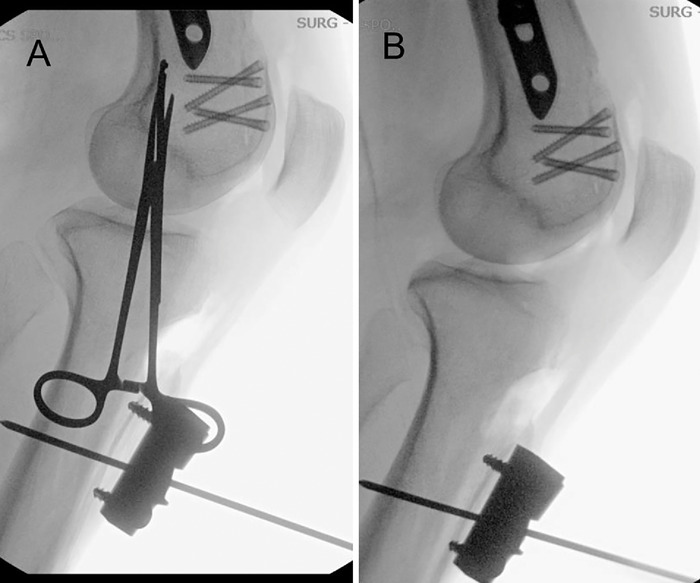
(A) The Schöttle point was localized fluoroscopically, a guide pin was placed, and an 8.5 × 30-mm tunnel was drilled obliquely, avoiding previous hardware. (B) Prior to transposition of the distal primary wedge, 15 mm of bone was resected, allowing distal displacement of the primary wedge with preliminary pin fixation and continued presence of the MD3T guide (Kinamed, Inc).

The medial quadriceps tendon–femoral ligament reconstruction entailed localizing the Schöttle point fluoroscopically, placing a guide pin, and drilling an 8.5 × 30-mm tunnel obliquely, avoiding previous hardware (Figure 8A). An 8.0 × 30-mm loop of fresh-frozen posterior tibialis tendon allograft (MTF Biologics) was fixed with an 8 × 19.5-mm tenodesis screw (Arthrex, Inc). The graft was then passed under the medial retinacular structures and through 2 vertical splits made in the quadriceps but were not tensioned prior to distal tibial tubercle osteotomy fixation.

**Figure 9.**
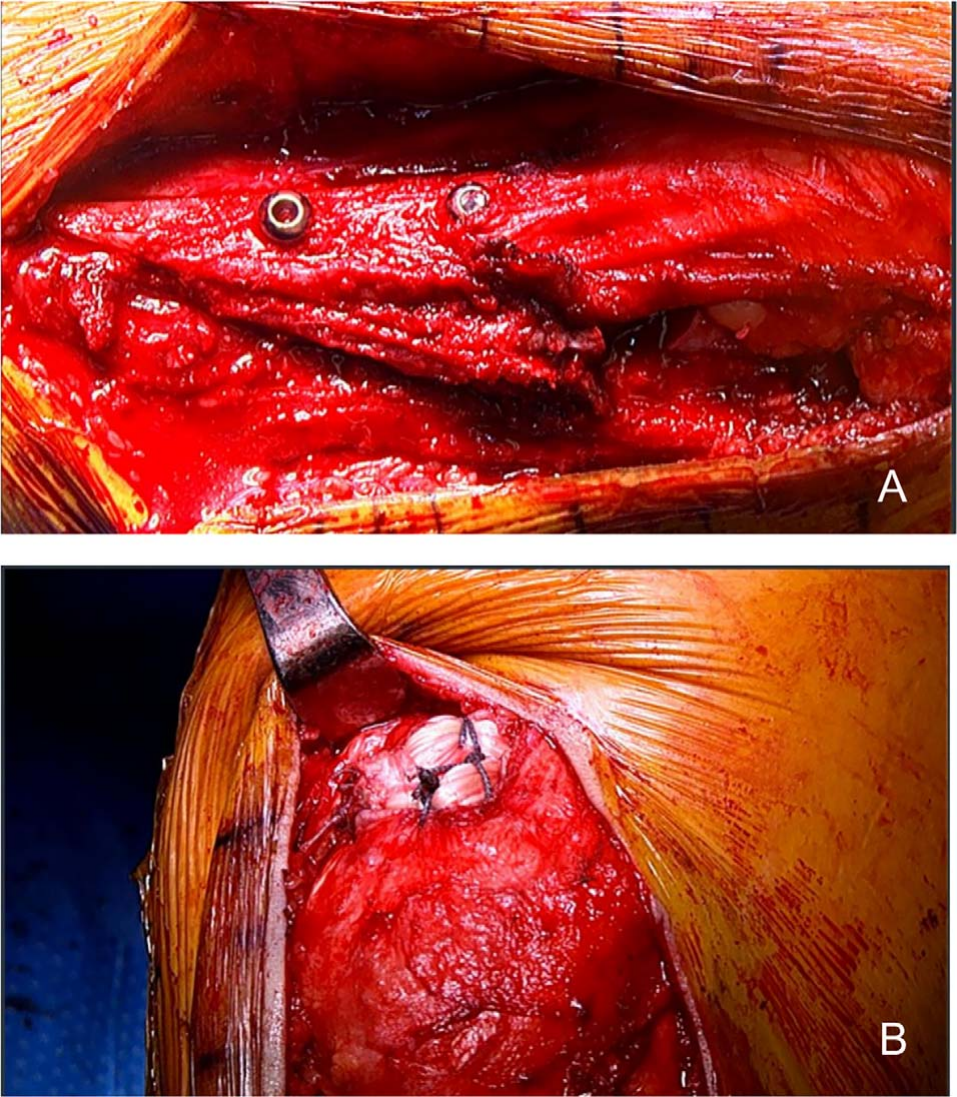
Intraoperative photographs show (A) the tibial tubercle osteotomy with transposition of wedges for medialization and distal displacement of the tubercle, with screw fixation using the lag screw technique, and (B) the medial quadriceps tendon-femoral ligament suture fixation using #2 Orthocord suture material (Depuy Mitek, Inc).

Transposition of the previous tibial tubercle wedges was then performed, medializing the primary distal tubercle 10 mm. Prior to transposition of the distal primary wedge, 15 mm of bone was resected, allowing distal displacement of the primary wedge at this length (Figure 8B). Fixation was performed using the standard lag screw technique with two 4.5-mm screws (DePuy Synthes) (Figure 9A). Autologous bone graft from the proximal wedge resection was impacted around the tibial tubercle osteotomy site to augment healing. The posterior tibialis tendon allograft was tensioned medially with the knee at 30° flexion, and tension was maintained by placing sequential figure-of-8 sutures using #2 Orthocord (Depuy Mitek, Inc) (Figure 9B). Central tracking of the patella was verified, and final fluoroscopic images were obtained. Standard closure was performed.

### Postoperative Course After Left Knee Surgery

**Figure 10.**
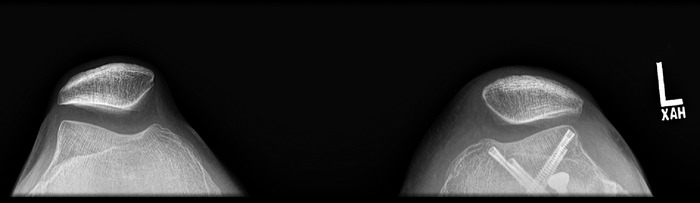
Merchant view radiograph of the left knee shows postoperative alignment following trochleoplasty.

Postoperatively, the patient's left knee was placed into a hinged immobilizer (T Scope, Breg, Inc) locked in –10° hyperextension and allowed flexion to 50° at rest. The patient progressed to full weight-bearing as tolerated with hinged immobilizer locked in extension for 6 weeks. Range of motion was sequentially increased to 90° by 4 weeks, with full flexion and discontinuation of the immobilizer allowed at 6 weeks. Radiographs demonstrated stable left tibial tubercle osteotomy and trochleoplasty. The patient demonstrated steady postoperative improvement in pain, function, and PROMs for the left knee. From 6 weeks to 3 months, pain severity decreased, and knee-specific, functional, and quality-of-life measures improved. By 6 months, the patient exhibited 0° extension and 140° flexion in the left knee with stable postoperative radiographs (Figure 10), along with continued gains in functional and sports-related outcomes, indicating ongoing recovery and improved knee performance. Left knee pain frequency, pain severity, and other PROMs recorded at the postoperative follow-ups are provided in Table 1.

### Right Knee Surgery

**Figure 11.**
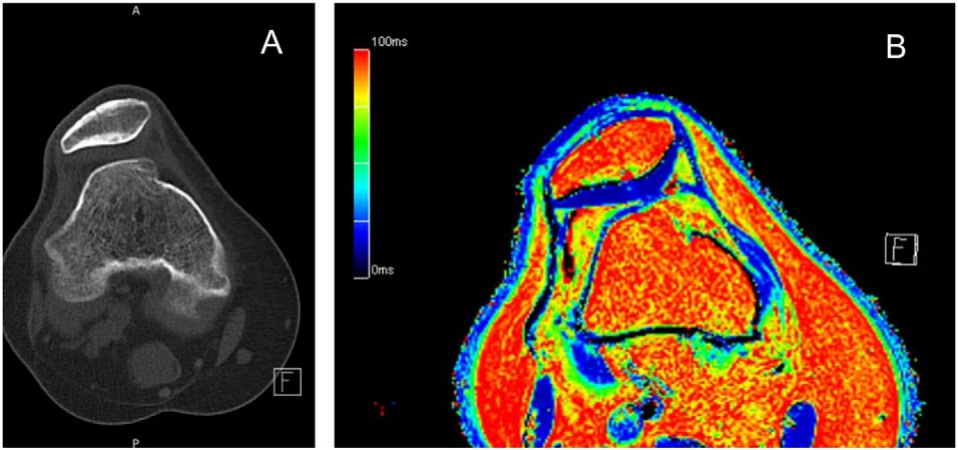
(A) Computed tomography scan of the right knee shows lateral patellar subluxation and trochlear Dejour type D dysplasia. (B) T2 mapping magnetic resonance imaging of the right knee shows minimal articular cartilage damage but continued lateral patellar subluxation.

**Figure 12.**
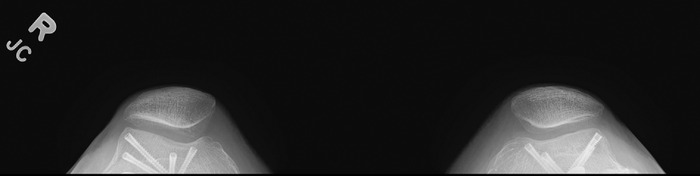
Merchant view radiograph shows postoperative alignment of both knees following bilateral trochleoplasty procedures.

The patient elected to undergo the same surgery for the right knee 12 months after the left knee surgery. Preoperative computed tomography (CT) and MRI studies of the right knee demonstrated severe lateral patellar subluxation and minimal patellofemoral articular cartilage damage (Figures 11A and 11B). The same postoperative protocol was followed for the right knee without any notable complications. Radiographs at 6 weeks postoperatively showed stable trochleoplasty, tibial tubercle osteotomy, and medial quadriceps tendon–femoral ligament procedures (Figure 12).

### Postoperative Course After Right Knee Surgery

Postoperatively, the patient demonstrated progressive bilateral improvement in pain, range of motion, and functional outcomes. Early follow-up of the right knee showed advanced range of motion with persistent pain and functional limitations. By 14 months after left knee surgery and 10 weeks after right knee surgery, the patient had regained full bilateral extension and flexion. The patient reported complete resolution of pain in the left knee with normalization of knee-specific and quality-of-life PROMs, whereas the right knee had moderate residual symptoms with improving functional scores. Preoperative and postoperative right knee PROMs are provided in Table 2.

**Table 2.** Patient-Reported Outcome Measures for the Right Knee

					Knee injury and Osteoarthritis Outcome Score (KOOS)					Short Form Health Survey	
Time Point	Pain Frequency^a^	Pain Severity^b^	IKDC Function^c^	Lysholm^d^	Pain^e^	Symptom^f^	ADL^g^	Sport^h^	QoL^i^	PSF-12^j^	MSF-12^k^
2 weeks preoperatively	5	4	66.66	75	69.44	78.57	97.06	70	43.75	31.49	60.73
6 weeks postoperatively	8	6	34.48	38	44.44	60.71	57.35	20	37.5	27.77	62.57
10 weeks postoperatively	5	4	50.57	69	58.33	75	75	45	56.25	34.97	60.52
5 months postoperatively	8	4	56.32	74	69.44	60.71	79.41	55	68.75	47.26	60.26
11 months postoperatively	6	3	74.71	85	83.33	75	97.06	75	68.75	53.8	57.92

^a^Pain frequency was subjectively scaled from 0 to 10, with 0 being never and 10 being constant.

^b^Pain severity was subjectively scaled from 0 to 10, with 0 being no pain and 10 being the most severe pain.

^c^The International Knee Documentation Committee (IKDC) form is scored from 0 to 100, with higher scores indicating better knee function and lower symptoms. A score of 100 indicates no limitations.

^d^The Lysholm Knee Scoring Scale is scored from 0 to 100, with higher scores indicating better knee function and fewer symptoms. It is an 8-item, patient-reported outcome measure assessing limp, support, locking, instability, pain, swelling, stair climbing, and squatting, with scores typically categorized as poor (<65), fair (65-83), good (84-94), and excellent (95-100).

^e^The KOOS pain outcome is scored from 0 to 100, with higher scores indicating better function and less knee-related pain.

^f^The KOOS symptom outcome is scored from 0 to 100, with higher scores indicating better function and fewer knee-related symptoms.

^g^The KOOS activities of daily living (ADL) outcome is scored from 0 to 100, with higher scores indicating better function and fewer knee-related problems with activities of daily living.

^h^The KOOS sport outcome is scored from 0 to 100, with higher scores indicating better function and fewer knee-related problems with sporting activities.

^i^The KOOS quality of life (QoL) outcome is scored from 0 to 100, with higher scores indicating better function and fewer knee-related problems impacting quality of life.

^j^The Short Form Health Survey physical component score (PSF-12) is scored from 0 to 100, with higher scores indicating better physical health status. Scores are norm-based (mean of 50, standard deviation of 10 in the general population), with values above or below 50 representing better or worse health relative to population norms.

^k^The Short Form Health Survey mental component score (MSF-12) is scored from 0 to 100, with higher scores indicating better mental health status. Scores are norm-based (mean of 50, standard deviation of 10 in the general population), with values above or below 50 representing better or worse health relative to population norms.

### Long-Term Follow-Up

**Figure 13.**
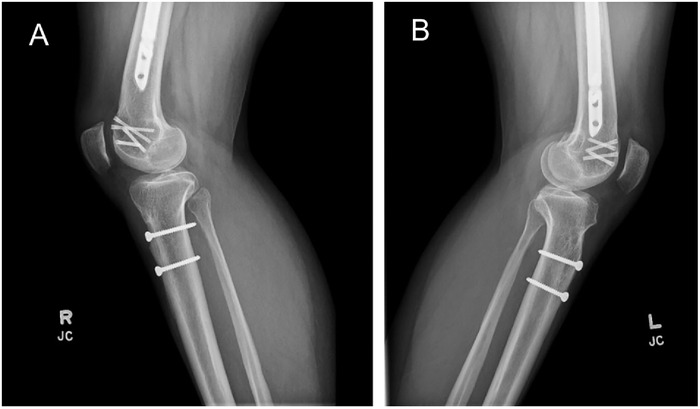
Lateral radiographs show (A) right knee 11 months postoperatively, and (B) left knee 2 years postoperatively.

Five months following right knee surgery, the patient reported unrestricted participation in daily activities, supported by stable radiographic findings and continued PROM improvement. At her most recent follow-up (11 months after right knee surgery, and 2 years after left knee surgery), the patient reported no recurrent patellar instability, demonstrated healed osteotomies and normal patellofemoral alignment on imaging (Figure 13), and maintained excellent left knee outcomes with continued functional improvement of the right knee with mild residual pain.

Overall, PROMs demonstrated an expected early postoperative decline followed by steady, progressive improvement across pain, function, and activity domains, culminating in sustained functional gains and high patient satisfaction at long-term follow-up.

## DISCUSSION

This case is unique because of the coexistence of severe Dejour type D trochlear dysplasia with multiple anatomic risk factors, including the patient's “miserable malalignment” (as described by the treating surgeon) that necessitated a comprehensive, cartilage-preserving surgical strategy rather than isolated soft-tissue stabilization. The case describes an innovative option for treating patellar instability caused by trochlear dysplasia recalcitrant to traditional procedures and highlights the effectiveness of a staged, stepwise surgical approach using a recession trochleoplasty technique that results in durable functional recovery.

The initial physical examination is a crucial step in assessing a patient after an episode of instability. Lower limb alignment, hypermobility, and gait should all be assessed, as well as the quadriceps (Q) angle and J-sign.^17^ A Q-angle measurement >20° in females and >15° in males may indicate a greater risk for patellar dislocation and should be interpreted within a comprehensive clinical assessment.^17,18^ A positive J-sign, described as the patella moving laterally as the knee is actively extended from a flexed position, is an important aspect of the physical examination in patients with recurrent lateral patellofemoral instability.^19^ X-ray, MRI, and CT scan are used to visualize bony abnormalities, including an elevated tibial tubercle–trochlear groove distance greater than 16 ± 4 mm in extension or 9 ± 4 mm in flexion.^20,21^ Other clinical clues include femoral anteversion (inward-pointed toes), medial-sided tenderness, and the presence of a large hemarthrosis or effusion.^2^ Several methods have been designed to measure patella alta, but 2 of the most reliable are the Insall-Salvati ratio and the Caton-Deschamps index.^22,23^ Many clinicians have moved to the use of the Caton-Deschamps index as we did in our case.

Physiotherapy techniques such as bracing, stretching, and neuromuscular exercises are commonly recommended as conservative treatment modalities.^24^ While surgical treatment is associated with a lower rate of redislocation compared to conservative measures, adequately powered, randomized, multicenter studies are required to determine if surgical management is truly superior.^13,24^ Previous literature favors conservative management for acute, primary dislocations with special considerations given in cases of osteochondral fragments and major defects of the parapatellar ligament complex.^25^ Duthon described cases of recurrent dislocations that mandated surgery because the severe apprehension of patients constituted a disability in everyday life and also because surgery would prevent chondral lesions that would cause patellofemoral osteoarthritis in the long term.^20^ Demey et al discussed the algorithm created for the treatment of patellar instability and the criteria for selecting conservative treatment vs surgical techniques such as arthroscopy, medial patellofemoral ligament reconstruction, and tibial tubercle lengthening or shortening.^26^

For patients with patellar instability in the setting of trochlear dysplasia, additional factors should be considered when determining appropriate treatment. Trochlear dysplasia can be difficult to diagnose and treat because of biomechanical and kinematic changes that often require surgical correction when symptomatic.^27^ As noted previously, the Dejour classification assesses the severity of trochlear dysplasia based on abnormal trochlear morphology seen on lateral knee radiographs and axial CT or MRI imaging.^7^ Classification of the type of trochlear dysplasia offers clinicians guidance on the appropriate surgical technique. Dejour types B and D, the presence of a J-sign, and history of recurrent dislocations have been reported as indications to consider trochleoplasty.^28-30^ Ammann et al reported that adolescent patients with recurrent patellar dislocations had higher knee flexion angles and knee extensor moments (comparable to a normal gait) after bilateral trochleoplasty, while patients undergoing unilateral trochleoplasty did not achieve a normal walking gait.^31^

The 3 categories of trochleoplasty procedures are lateral facet elevation, subchondral deepening, and recession wedge techniques.^32^ In patients with Dejour types B and D trochlear dysplasia, a supratrochlear spur is present, and wedge resection addresses this deformity. Goutallier et al first described this technique in 2002, and the approach was verified by Thaunat et al in 2011.^33,34^ The patella must pass over the spur which exhibits a bony mound morphologically. Femoral rotational osteotomy, while addressing one component of the patient's “miserable malalignment,” did not correct the factor that contributes the most to the J-sign. Tibial tubercle osteotomy alone will not correct the problem, as medial and distal displacement of the tubercle does not correct the bone prominence that impacts patellar movement when the knee is flexed from an extended position. Once again, the J-sign is not addressed. Similarly, medial patellofemoral ligament and medial quadriceps tendon–femoral ligament procedures will not correct this problem.

However, the complexity and the complications associated with trochleoplasty give surgeons pause when they contemplate using this procedure. In a systematic review and meta-analysis of 1,000 trochleoplasties, recurrent dislocation occurred in 2.4% (24/994); the Dejour deepening trochleoplasty was most effective with only 1 recurrence in 349 knees (0.28%).^35^ Residual patellar instability without dislocation occurred in 82 of 754 knees (for a combined rate of 8% [95% CI 3%-14%]), patellofemoral osteoarthritis occurred in 117 of 431 knees (27%), stiffness occurred in 59 of 642 knees (for a combined rate of 7% [95% CI 3%-12%]), and subsequent surgery was required in 151 of 904 knees (16.7%).^35^

In our case, care was taken to avoid damage to the articular cartilage while screws were positioned peripherally and countersunk at the margins. Because of the patient's recurrent patellar instability despite bilateral femoral osteotomies, additional procedures were performed to augment the stability provided by the trochleoplasties.

Tibial tubercle distalization via osteotomy is a surgical technique with good outcomes that can be personally tailored to patients who experience recurrent patellar dislocations and have failed previous therapies.^36^ Medial quadriceps tendon–femoral ligament reconstruction is an alternative surgical technique to medial patellofemoral ligament reconstruction for patients with patellar instability. Medial quadriceps tendon–femoral ligament reconstruction requires no patellar drill hole, thus eliminating iatrogenic fracture risk while providing reliable patellofemoral joint stability.^37^ In our patient, medial quadriceps tendon–femoral ligament reconstruction was used to augment stabilization with minimal additional morbidity to the patient or additional time added to the procedure.

## CONCLUSION

Recession wedge trochleoplasty can effectively address chronic patellar instability associated with Dejour type D trochlear dysplasia in the setting of elevated tibial tubercle–trochlear groove distances, patella alta, and femoral anteversion. Additional procedures can maximize functional stability as demonstrated in this complex patient and permit a healthy, active lifestyle. Early intervention with meticulous preservation of the native articular cartilage is critical. Maintaining cartilage integrity may mitigate the risk of progressive degeneration and subsequent osteoarthritis. Care should be taken to avoid damage to the intact articular surface, thereby potentially avoiding later osteoarthritis.

## Notes

*This article meets the Accreditation Council for Graduate Medical Education and the American Board of Medical Specialties Maintenance of Certification competencies for Patient Care and Medical Knowledge.*
